# Data of preparation and characterization of activated carbon using two activant agents and mango seed as precursor material

**DOI:** 10.1016/j.dib.2019.104769

**Published:** 2019-11-06

**Authors:** Oscar D. Caicedo-Salcedo, Diana P. Vargas-Delgadillo, Liliana Giraldo, Juan Carlos Moreno-Piraján

**Affiliations:** a Facultad de Ciencias, Departamento de Biología, Grupo de Investigación en Materiales Porosos con Aplicaciones Ambientales y Tecnológicas, Universidad del Tolima, Colombia; b Facultad de Ciencias, Departamento de Química, Grupo de Investigación en Materiales Porosos con Aplicaciones Ambientales y Tecnológicas, Universidad del Tolima, Colombia; c Facultad de Ciencias, Departamento de Química, Universidad Nacional de Colombia, Bogotá, Colombia; d Facultad de Ciencias, Departamento de Química, Grupo de Investigación en Sólidos Porosos y Calorimetría, Universidad de los Andes, Bogotá, Colombia

**Keywords:** Activated carbon, Mango, Chemical activation, Characterization

## Abstract

The aim of this research is to prepare various carbonaceous materials with different textural, structural and chemical characteristics, using mango seed a rarely used residue for the preparation of activated carbons, as the precursor material. The mango seed was analyzed by TGA and SEM also methodological data about the preparation of activated carbons are provided. Four activated carbons were prepared using sulfuric acid (H_2_SO_4_) and calcium chloride (CaCl_2_) as activating agents and were characterized by means of TGA, SEM/EDX, Boehm Titration, isotherm determination of N_2_ adsorption-desorption at −196 °C and immersion calorimetry. Four carbons were obtained with superficial areas BET between 6 and 33 m^2^ g^−1^ and different chemical characteristics associated with the changes in the concentration of the activating agents. The activated carbons that were prepared with the highest activating agent concentrations, obtained better results in the amount of oxygenated surface groups, the total acidity and the amount of fixed carbon. The enthalpy of immersion in water was between 7 and 16 J g^−1^.

**Table:** Specifications Table

Subject area	Chemistry
More specific subject area	Material Science: Actived Carbon
Type of data	Table, images, text, graphs and figures
How data was acquired	TGA–DTA (Hitachi model 7200),SEM/EDX (JEOL JSM-6490LV; Tescan Lyra III), Isotherm determination of N_2_ adsorption-desorption at −196 °C (IQ2, Quantachrome Inc.), immersion calorimeter (“home-made”) and SCHOTT TritoLine Alpha plus TA20plus.
Data format	Raw, filtered and analyzed.
Parameters for data collection	The actived carbons were prepared using mango seed and two concentrations of calcium chloride solutions and two concentrations of sulfuric acid solutions were used to establish the incidence of the concentration of the activating agent on the textural, structural and chemical characteristics.
Description of data collection	The activated carbons were prepared from mango seed, which was collected in a pulping factory. These seeds were cleaned and dried at 90 °C. Subsequently, the seeds were crushed and their particle size was reduced to a size no greater than 2 cm. The three parts of the seed were mixed in order to obtain samples of that mixture. The precursor material was characterized by means of scanning electron microscopy (SEM) and a thermogravimetric analysis (TGA). Two activant agents, sulfuric acid and calcium chloride were used for their activation; concentrations of 5% and 15% v/v were used for the sulfuric acid and 3% and 7% w/v were used for the calcium chloride. The materials obtained were characterized structurally, texturally and chemically.
Data source location	Facultad de Ciencias, Departamento de Química, Universidad de los Andes, Bogotá, Colombia. Facultad de ciencias, Departamento de Química, Universidad Nacional De Colombia, Bogotá, Colombia.
Data accessibility	With the article

**Table:** 

**Value of the Data** • The termogravimetric analysis gives important data about the precursor material. • The detailed characterization, chemical and structural, is very useful for analyzing the applications of these materials. • The changes in the concentrations of the activation agent in the preparation of activated carbons allow the scientific analysis of the different solids could be obtained with different precursors. • Immersion calorimetry provides important enthalpic data that could be relationated with the textural and chemistry characteristics of the materials.

## 1 Data

**Fig. 1:**
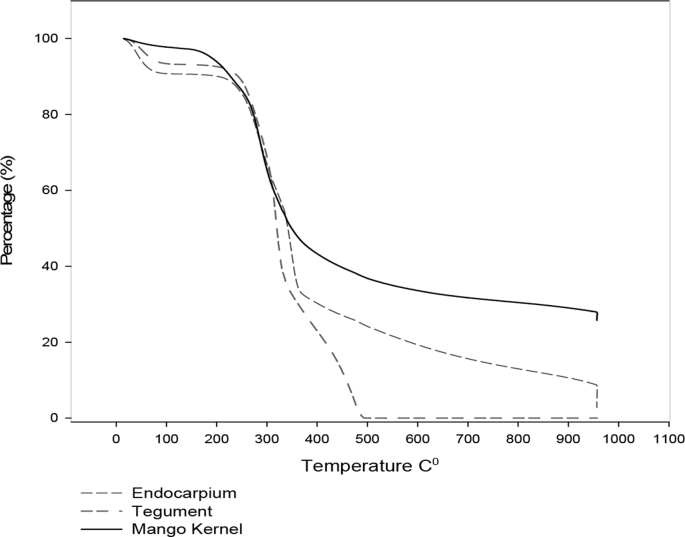
Termogravimetric analysis of mango seed parts.

**Fig. 2:**
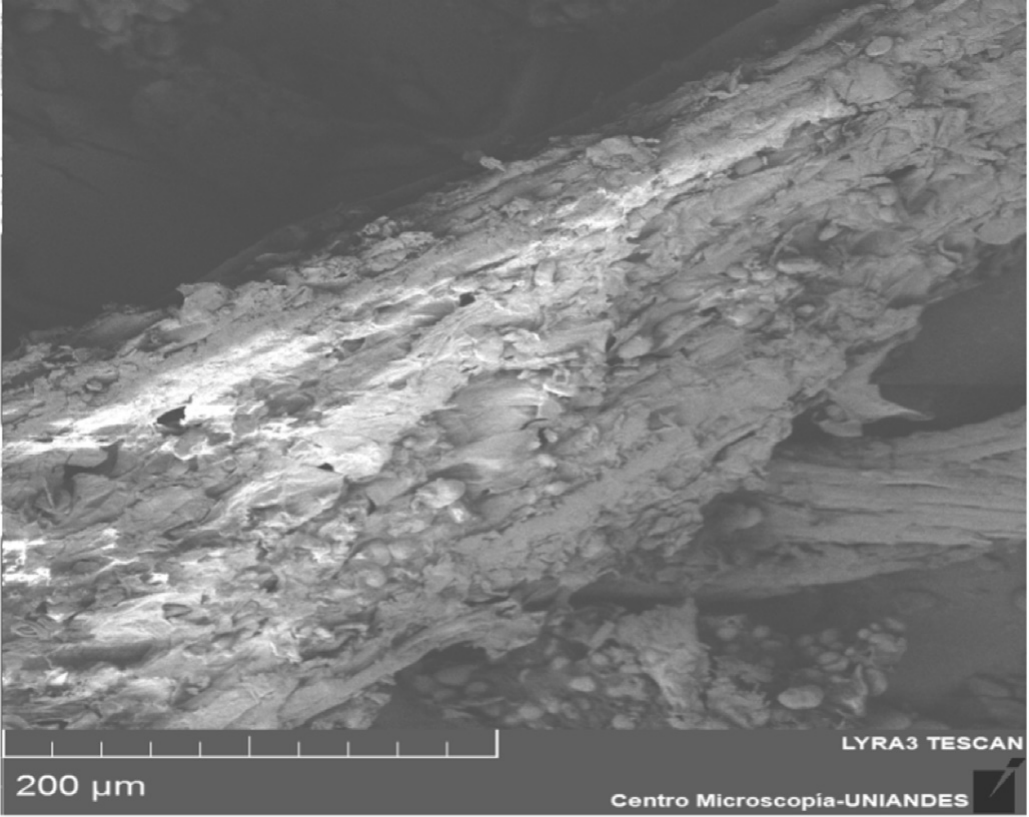
Mango seed microphotography.

**Fig. 3:**
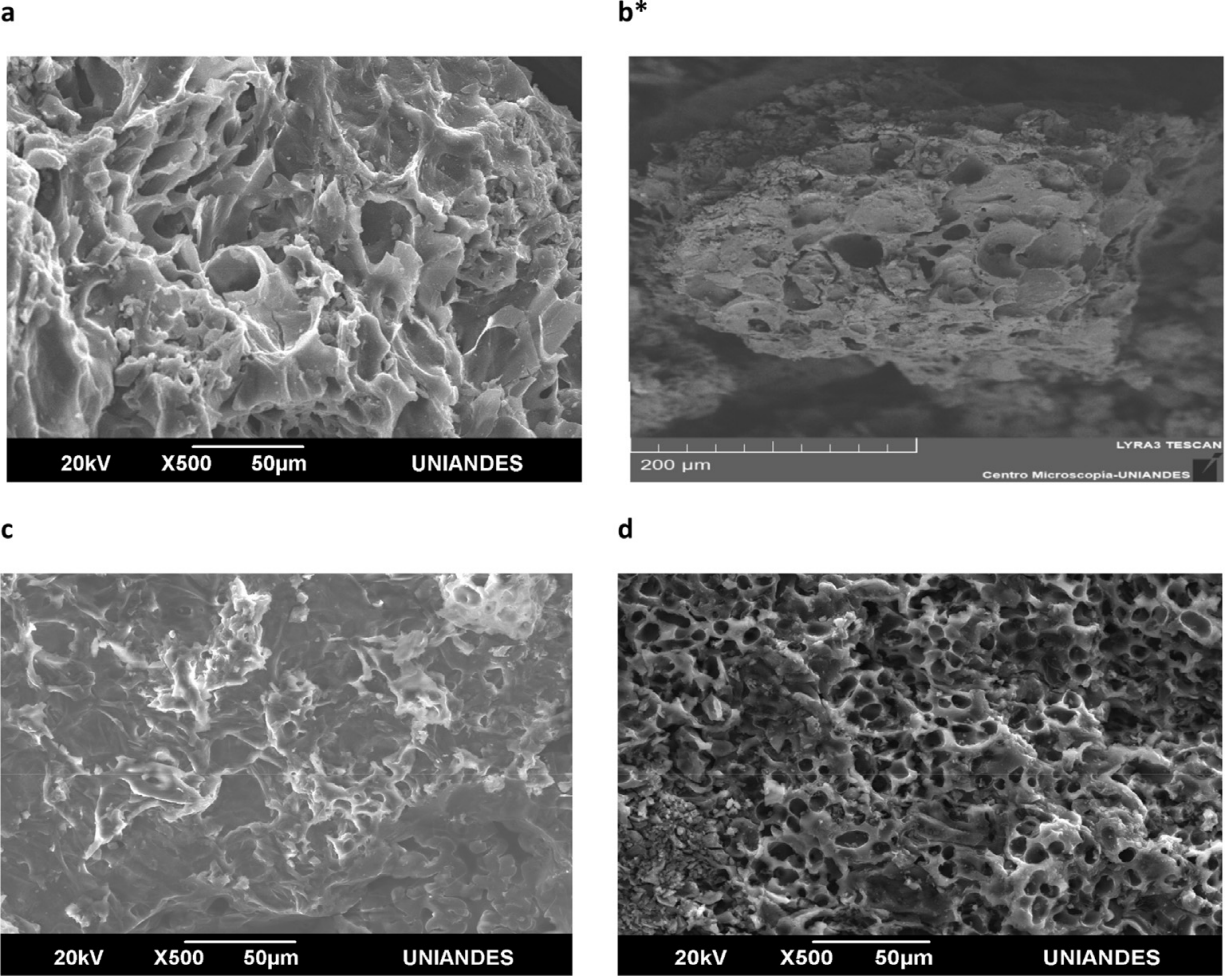
SEM images a) CAC3 b) CAC7*c) CAS5 d) CAS15. *The microphotography was taken with Tescan Lyra III microscope.

**Fig. 4:**
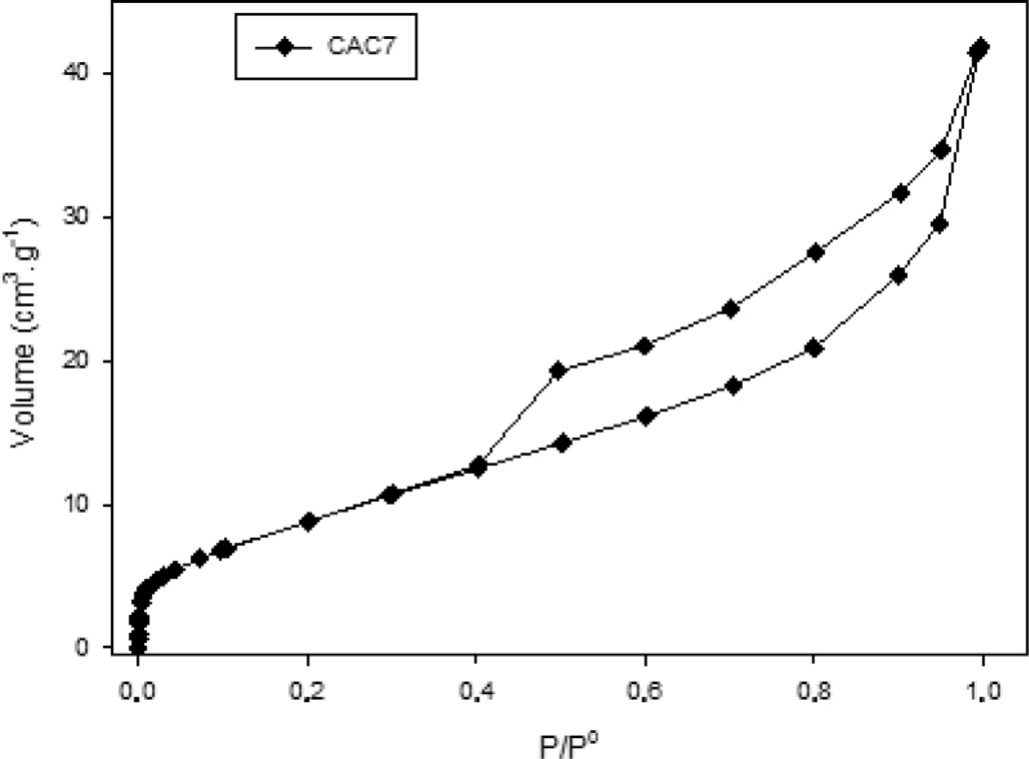
Adsorption isotherms of nitrogen on activated carbon **(CAC7)** at −196 °C.

**Fig. 5:**
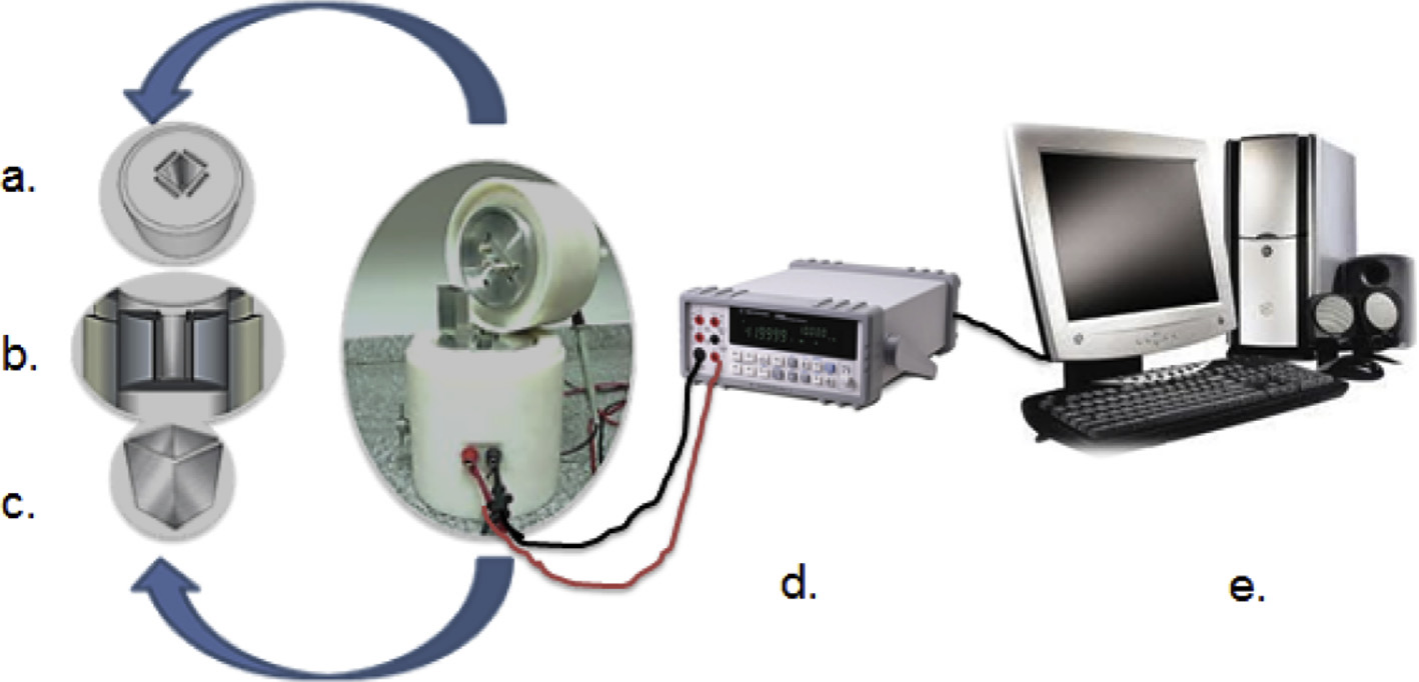
Immersion calorimeter used in this research. a-b) Cell socket view c) calorimetric cell d)Multimeter e)computer [[4], [5], [6]].

**Fig. 6:**
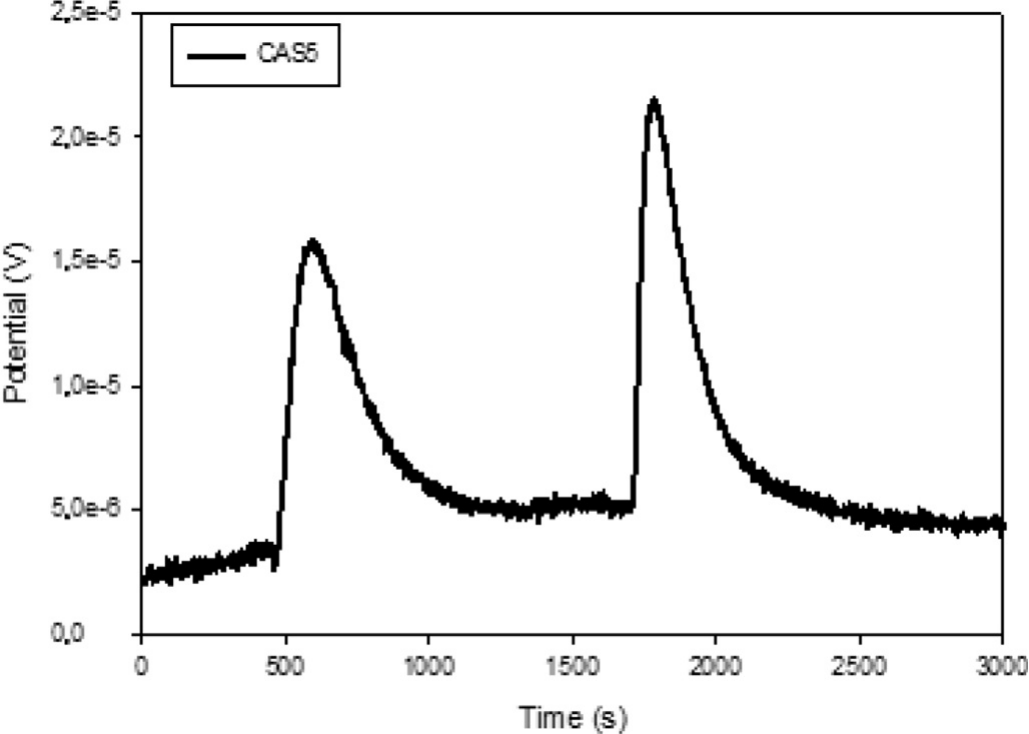
Calorimetric curve of the immersion of CAS5 into distilled water.

Different characterization data are given in this paper; the termogravimetric analysis is presented in Fig. 1, the Scanning electron microscope (SEM) shows an image of the precursor surface in Fig. 2 and also shows images of the surface of the carbons obtains in Fig. 3. Table 1 shows different values obtain in the proximate analysis, given data about the difference between each activated carbon. EDX data are shown in Table 2. Superficial area data are shown in Table 3, also Fig. 4 shows adsorption isotherm of nitrogen on CAC7 at −196 °C. The data of the characterization of superficial chemistry is shown in Table 4. Table 5 shows the values of enthalpy of immersion of the activated carbons. In Fig. 5, the homemade calorimeter used in this research it's shown. Fig. 6 shows the calorimetric curve of the immersion of CAS5.

**Table 1:** Proximate analysis of activated carbon obtain of mango seed.

Sample	Moisture	Volatile matter	Ash	Fixed Carbon
CAC3	13	33.6	5.1	48.3
CAC7	8.2	29	0.9	61.9
CAS5	6.2	31.8	0.3	61.7
CAS15	8.2	32	1.4	58.4

**Table 2:** Quantitative distribution of the elements present in the actived carbons surface determined by EDX.

Sample	C % Total weight	O % Total weight	S % Total weight	Ca % Total weight
CAC3	69.14	15.9	–	14.96
CAC7	65.61	26.75	–	7.64
CAS5	66.62	32.70	0.68	–
CAS15	72.70	18.94	8.36	–

**Table 3:** Analysis of S_BET_.

Sample	S_BET_	V_0_
	(m^2^.g^−1^)	(cm^3^.g^−1^)
CAC3	12	0.007
CAC7	33	0.01
CAS5	6	0.003
CAS15	12	0.006

**Table 4:** Surface chemistry characterization (Boehm Tritation).

Sample	Carboxylic groups (mmol.g^−1^)	Lactone groups (mmol.g^−1^)	Phenolic groups (mmol.g^−1^)	Total acidity (mmol.g^−1^)	Total basicity (mmol.g^−1^)	pH/PZC(Point Zero Charge)
CAC3	0.137	0.089	1.414	1.462	0.139	7.2
CAC7	0.305	1.318	3.852	2.840	1.739	6.7
CAS5	0.352	0.154	1.478	1.676	0.056	5.0
CAS15	0.500	0.219	2.142	2.424	0.365	5.4

**Table 5:** Values of enthalpy of immersion of the activated carbons obtain into distilled water.

Sample	-ΔH_im_ H_2_O (J g^−1^)
CAC3	10.77
CAC7	16.06
CAS5	7.209
CAS15	8.371

## 2 Experimental design, materials and methods

### 2.1 Materials

A large number of mango seeds were collected in a fruit pulp factory. The calcium chloride was purchased from Merck^tm^, also the sulfuric acid was purchased from Merck^tm^.

### 2.2 Preparation of the activated carbon

Different tropical countries have high mango (*Mangifera Indica*) production. Colombia produces more than 260.000 tons of mango per year. The mango seed has no commercial value and is a common residue of the industrial processes to obtain mango pulp [1]. Thus, the mango seed has potential as a precursor material for the elaboration of activated carbons due to its characteristics as lignocellulosic material, this is supported by different scientific reports that have demonstrated its versatility in terms of the textural and chemical characteristics that can be obtained by using a particular activating agent [[2], [3], [4]]. In that way, different types of activating agents have been used for the preparation of activated carbons. Several data reports show that calcium chloride allows to obtain materials with mesoporous characteristics and the concentration of calcium can directly affect the carbonaceous matrix [5]. In other way, acid activating agents, such as phosphoric or sulfuric acid, are related to generate high microporosity and a much stronger attack on the lignocellulosic structure of the precursor [2,5,6]. Two concentrations were used for each one of the activating agents, looking for a point of contrast in terms of the concentration of the activant and the characteristics given to the activated materials [5,6]. The mango seed was collected in a fruit pulp processing plant and cleaned before impregnation. The seed was dried in an oven at 90 °C and then crushed to obtain a mixture of the three parts of the seed (endocarp, tegument and kernel). Four samples of the mixture were taken and impregnated with each one of the raised solutions during 48 hours. The carbonization was carried out in a transverse furnace in N_2_ atmosphere with a flow of 80 cm^3^.min^−1^ with a heating ramp of 10 °C min^−1^, up to a temperature of 450 °C, having a residence time of 2 h. Each of the samples was washed with distilled water until a constant pH was obtained.

The samples obtained were called as:

CAC3 (activated carbon with calcium chloride solution 3% p/v).

CAC7 (activated carbon with calcium chloride solution 7% p/v).

CAS5 (activated carbon with sulfuric acid solution 5% v/v).

CAS15 (activated carbon with sulfuric acid solution 15% v/v).

### 2.3 Experimental desing

#### 2.3.1 Mango seed data

Thermogravimetric analysis was performed on each of the parts that make up the mango seed, the data are shown in Fig. 1 The loss of mass between the different parts of the seed is considerable; the mango kernel had the least loss of mass. However, the tegument loses all its mass when reaching temperatures close to 500 °C.

The mango seed were characterized by SEM microscopy, given data about the structure it has in the surface (Fig. 2). The microphotography shows a fibrous structure with little or no porous surface, characteristic of the lignocellulosic materials.

#### 2.3.2 Activated carbon characterization data

The four materials obtained were characterized; textural, structural and chemical characterization data are given below. The proximate analysis gives important information about the composition of the activated carbon. In Table 1 data are shown. CAC3 has the lowest percentage of fixed carbon, this relationated with the low attack of the activant agent; also it has the highest percentage of ash of all the carbons.

The two materials that were activated with the highest concentration, (CAC7-CAS15) have similar moisture, but has difference in the fixed carbon and the volatile matter. Although CAS5 has the lowest percentage of ash it has one of the highest percentage of fixed carbon.

##### 2.3.2.1 SEM characterization

The SEM images show important data of the difference between the activante agents. The CAS15 microphotography shows a huge attack in the surface, generating a lot of macroporous (Fig. 3).

Despite the difference in the concentration, the activant agents done a good work attacking the surface and the lignocelluloses structure, also proving different superficial chemistry (Table 4). In addition, the EDX results are shown in Table 2.

The data reported by the EDX analysis gives important information about the chemical surface composition of the materials. As can be seen in Table 2, the carbons that were activated with calcium chloride reported the presence of calcium on their surface. Moreover, the materials that were activated with sulfuric acid, obtained the presence of sulfur on its surface.

##### 2.3.2.2 Isotherm determination of N_2_ adsorption-desorption at −196 °C

Fig. 4. Shows the adsorption – desorption isotherms of N_2_ at −196 °C for **CAC7** which obtained the largest surface area (33 m^2^ g^−1^). This isotherm is type IV and the presence of the type 3 hysteresis cycle is easily observed [7].

The data obtained demonstrate low surface areas for all materials. Calcium chloride generated greater surface areas being 12 and 33 m^2^ g^−1^ for materials CAC3 and CAC7. On the other hand, the activation with sulfuric acid generated lower areas of the order of 6 and 12 m^2^ g^−1^, for CAS5 and CAS15 respectively.

Table 4 shows the data obtained from the chemical characterization of the materials, where changes are observed in the content of surface groups with respect to the activating agent and the concentrations that were used.

The materials that were activated using the highest concentrations, obtained greater changes in their surface chemistry, presenting a greater number of surface groups, both carboxylic and phenolic, this data are important to show the difference between two activant agents with a non-common precursor [[2], [3], [4], [5]]. The two carbons that were activated with sulfuric acid, obtained an acid point of zero charge, on the other hand, the materials activated with calcium chloride obtained a charge point closer to neutral. The presence of a great variety of oxygenated groups like phenolic and carboxylic could suggest the use of these activated carbons for the adsorption of certain metal ions in aqueous solution.

##### 2.3.2.3 Immersion calorimetry of activated carbon in distilled water

The four activated carbons obtained were immersed in distilled water using a Tian-type calorimeter Fig. 5 that was built in our laboratory. The intensity of the interaction between the solution and the activated carbon can be determined by means of the enthalpy of immersion of the solid in solutions containing active substances or in this case water. The immersion calorimetry in water, allows to relate the content of oxygenated groups in the surface of the materials and their specific interaction with the polar molecules of water. This technique allows to know the energy that is manifested in the surface as well as relating its results with other data obtained from the chemical characteristics of the materials.

The procedure to obtain the enthalpy of immersion consists of placing 10 ml of solvent in a metal cell Fig. 5 (c). In the heat reservoir of the calorimeter at 298K; the calorimeter registers the output of electric potential as long as it has reached a baseline over time. Then, 100mg of the activated carbon sample is placed in a glass vial and the immersion is performed [8,9]. If the immersion was satisfactory and the baseline is obtained again, the electrical calibration is performed. The variation of the electric potential versus time can be used for the calculation of the immersion enthalpy [8,9]. Fig. 6 shows the data obtain; the first observed peak corresponds to the immersion process, when the solid get contact with the solvent, the second one corresponds to the electrical calibration of the calorimeter.

The data of the immersion enthalpies of the materials obtained are shown in Table 5. The materials that were activated with calcium chloride (CAC3-CAC7) obtained the higher values in the immersion calorimetry; otherwise the materials that were activated with sulfuric acid got the lowest values; these values can be relationated with the oxygen groups in the surface of each material and also with the total acidity and basicity shown in Table 3, this data is important because it gives information about the several interactions between the surface of the carbons and different molecules, in this case with water.
